# Supplementary material for: Niche Evolution and Conservation of a Chinese Endemic Genus Sinojackia (Styracaceae)
Source: Biology (Basel). 2024 Dec 22;13(12):1085. doi: 10.3390/biology13121085 (PMC11673067; doi:10.3390/biology13121085)
Supplement: Supplementary file 1 [file biology-13-01085-s001.zip › biology-3340644-supplementary.pdf]

Table 1 The complete chloroplast sequences information used in this study.

Species	GenBank no.	References
Sinojackia huangmeiensis	MN694844	Dong et al., 2020
Sinojackia microcarpa	MW421726	Zhong et al., 2021
Sinojackia microcarpa	MW026412	Unpublished, www.ncbi.nlm.nih.gov/nuccore/MW026412
Sinojackia microcarpa	MG719835	Unpublished, www.ncbi.nlm.nih.gov/nuccore/MG719835
Sinojackia sarcocarpa	MT700476	Unpublished, www.ncbi.nlm.nih.gov/nuccore/MT700476
Sinojackia sarcocarpa	MT700477	Unpublished, www.ncbi.nlm.nih.gov/nuccore/MT700477
Sinojackia xylocarpa	MT700481	Unpublished, www.ncbi.nlm.nih.gov/nuccore/MT700481
Sinojackia rehderiana	MT700475	Unpublished, www.ncbi.nlm.nih.gov/nuccore/MT700475
Sinojackia sarcocarpa	MK351986	Fan et al., 2019
Sinojackia oblongicarpa	OQ985171	
Sinojackia oblongicarpa	OQ985172	Jian et al., 2024
Sinojackia oblongicarpa	OQ985173	
Sinojackia xylocarpa	MG719827	Unpublished, www.ncbi.nlm.nih.gov/nuccore/MG719827
Sinojackia xylocarpa	MH782178	Lin et al., 2019
Rehderodendron macrocarpum	NC_041139	Unpublished, www.ncbi.nlm.nih.gov/nuccore/NC_041139
Melliodendron xylocarpum	MN378563	Wang et al., 2019
Changiostyrax dolichocarpus	NC_041171	Unpublished, www.ncbi.nlm.nih.gov/nuccore/NC_041171
Perkinsiodendron macgregorii	NC_041136	Unpublished, www.ncbi.nlm.nih.gov/nuccore/NC_041136
Halesia diptera	NC_041128	Unpublished, www.ncbi.nlm.nih.gov/nuccore/NC_041128
Pterostyrax hispidus	NC_041135	Unpublished, www.ncbi.nlm.nih.gov/nuccore/NC_041135
Styrax wuyuanensis	MW166213	Unpublished, www.ncbi.nlm.nih.gov/nuccore/MW166213

References:

Dong, H., Wang, H., Li, Y. and Yu, J. 2020. The complete chloroplast genome sequence of *Sinojackia huangmeiensis* (Styracaceae). Mitochondrial DNA B Resources, 5, 715-717.

Fan, J., Fu, Q.C., Liang, Z. 2019. Complete chloroplast genome sequence and

phylogenetic analysis of *Sinojackia sarcocarpa*, an endemic plant in Southwest China. Mithochondrial DNA B Resources, 1350-1351.

Jian, X., Wang, Y.L., Li, Q., Miao, Y.M. 2024. Plastid phylogenetics, biogeography, and character evolution of the Chinese endemic genus *Sinojackia* Hu. Diversity 2024, 165, 305.

Lin, H.Y., Hao, Y.J., Li, J.H., Fu, C.X., Soltis P.S., Soltis D.E., Zhao Y.P. 2019. Phylogenomic conflict resulting from ancient introgression following species diversification in *Stewartia* s.l. (Theaceae). Molecular Phylogenetics and Evolution, 135, 1-11.

Wang,Y., Xu,X., Tong,L., Zhang,Y. and Zhao,Z. 2019. The complete chloroplast genome sequence of *Melliodendron wangianum* (Styracaceae). Mitochondrial DNA B Resources, 4, 4053-4054.

Zhong, T.L., Zhuo, J., Chen, D.W., Vasupalli, N., Chen, J.M., Qian QX. 2021. Complete chloroplast genome sequence of *Sinojackia microcarpa* (Styracaceae): comparative and phylogenetic analysis. Biologia, 76, 3891-3900.

Table S2. The occurrence records of *Sinojackia* used in the niche analysis.

No.	Latitude	Longitude
1	30.254875	120.116732
2	30.2386	119.7997
3	29.563729	119.570671
4	27.57	110.04
5	25.39996	112.948819
6	30.966552	111.261345
7	24.813781	113.184435
8	28.746779	115.826905
9	29.32	103.71
10	32.188833	118.700951
11	32.024776	118.973625
12	32.150475	118.706452
13	32.074711	118.964742
14	32.023	118.704
15	32.039152	118.924486
16	32.104451	118.933659
17	32.000166	118.96621
18	30.27	120.17
19	29.86	121.54
20	32.05	118.79
21	32.055645	118.797741
22	31.92228	119.174162
23	27.653	109.8705
24	29.537061	103.771587
25	29.829129	110.889869
26	26.722151	111.610857
27	30.512839	118.610857
28	24.778326	113.270591

Table S3. The filtered occurrence records of *Sinojackia* used in species distribution modeling.

No.	Latitude	Longtitude
1	30.25	120.1
2	30.25	119.71667
3	32.05	118.816666
4	30.533333	114.416664
5	32.150475	118.706452
6	32.023	118.704
7	32.039152	118.924486
8	32.104451	118.933659
9	32.000166	118.96621
10	30.27	120.17
11	29.86	121.54
12	25.39996	112.948819
13	30.966552	111.261345
14	24.813781	113.184435
15	28.746779	115.826905
